# Emerging public health strategies in malaria control: innovations and implications

**DOI:** 10.1097/MS9.0000000000002578

**Published:** 2024-09-20

**Authors:** Emmanuel Ifeanyi Obeagu, Getrude Uzoma Obeagu

**Affiliations:** a Department of Medical Laboratory Science, Kampala International University, Kampala, Uganda; b School of Nursing Science, Kampala International University, Kampala, Uganda

**Keywords:** innovations, malaria control, malaria vaccines, public health strategies, vector control

## Abstract

Malaria remains a significant global health challenge, particularly in regions with limited resources and tropical climates. Despite extensive efforts, the disease continues to cause significant morbidity and mortality, with ~229 million cases and 409 000 deaths reported in 2020. However, recent years have seen promising advancements in public health strategies aimed at malaria control and elimination. Technological advancements have played a crucial role in improving malaria control efforts. Genomic surveillance techniques enable the monitoring of malaria parasite populations, aiding in the detection of drug resistance and informing targeted interventions. Additionally, innovative diagnostic technologies, such as rapid diagnostic tests (RDTs) and molecular assays, have enhanced the speed and accuracy of malaria diagnosis, facilitated prompt treatment and reduced transmission. These tools are instrumental in achieving the WHO goals of reducing malaria cases and deaths by at least 90% by 2030. Novel vector control methods offer innovative approaches to reduce malaria transmission. Insecticide-treated nets (ITNs) and indoor residual spraying (IRS) remain foundational strategies, with advancements including the development of next-generation insecticides and long-lasting insecticidal nets (LLINs). Furthermore, genetic modification of mosquitoes, such as gene drive technology, holds promise for reducing mosquito populations and interrupting malaria transmission. These vector control innovations complement other strategies, contributing to comprehensive malaria control efforts aimed at achieving sustainable disease reduction and eventual elimination.

## Introduction

Highlights

- Technological innovations in malaria control: Recent advancements in genomic surveillance, rapid diagnostic tests, and novel treatment options are revolutionizing malaria control.
- Novel vector control methods: Innovative approaches such as genetic modification of mosquitoes, biological control agents, and spatial repellents offer new solutions.
- Policy implications: The integration of emerging malaria control strategies into national policies requires robust regulatory frameworks.
- Climate change adaptation: Climate change impacts malaria transmission.
- International Cooperation and Funding: Global collaboration and funding are vital for advancing malaria control innovations.

Malaria stands as one of the most formidable challenges in global public health, particularly in regions with tropical climates and limited resources^1^. Despite considerable progress over the past decades, the disease continues to exact a heavy toll on human health and well-being^2^. According to the WHO, an estimated 229 million malaria cases and 409 000 deaths were reported worldwide in 2020, with the majority occurring in sub-Saharan Africa^3^. Malaria disproportionately affects vulnerable populations, including children under five years of age and pregnant women, further exacerbating socio-economic disparities and hindering development efforts in endemic regions. The fight against malaria has been characterized by multifaceted approaches, ranging from vector control and case management to preventive measures and research into vaccines and novel interventions^4^. Historically, the deployment of ITNs, IRS, and antimalarial drugs has been pivotal in reducing malaria incidence and mortality^5^. However, the emergence of drug-resistant parasites and insecticide-resistant mosquitoes has underscored the need for innovative and sustainable strategies to combat the disease effectively.

Recent years have witnessed significant advancements in malaria control strategies, driven by technological innovations, scientific research, and collaborative efforts among governments, international organizations, and the private sector. These emerging strategies offer renewed hope for malaria control and elimination, aiming to reduce transmission, prevent disease outbreaks, and ultimately achieve the WHO’s ambitious goal of malaria eradication^6^. Technological innovations have revolutionized the landscape of malaria control, empowering researchers and public health practitioners with new tools and approaches to combat the disease^7^. Genomic surveillance, for instance, allows for the sequencing and analysis of malaria parasite genomes, enabling real-time monitoring of parasite populations and the detection of drug-resistance mutations^8^. This genomic data provides valuable insights into the dynamics of malaria transmission, guiding the deployment of targeted interventions and the development of more effective antimalarial drugs. In addition to genomic surveillance, advancements in diagnostic technologies have transformed malaria diagnosis and case management. Rapid diagnostic tests (RDTs) have become indispensable tools in resource-limited settings, providing quick and accurate detection of malaria parasites at the point of care^9^. Molecular diagnostic assays, such as polymerase chain reaction (PCR) tests and loop-mediated isothermal amplification (LAMP), offer enhanced sensitivity and specificity, particularly in low-transmission settings where traditional microscopy may yield false-negative results^10^. These diagnostic innovations enable prompt treatment initiation, reducing the risk of severe illness and interrupting malaria transmission chains. Vector control remains a cornerstone of malaria prevention efforts, with recent innovations expanding the toolkit available to public health authorities^11^. While ITNs and IRS continue to be widely used, next-generation insecticides and long-lasting insecticidal nets (LLINs) have been developed to address insecticide resistance and improve durability^12^. Moreover, advances in genetic engineering, including gene drive technology, hold promise for reducing mosquito populations and disrupting malaria transmission cycles. These novel vector control methods complement existing strategies, offering new avenues for achieving sustainable malaria control and elimination.

## Aim

The aim of this review is to comprehensively analyze and synthesize recent advancements in malaria control strategies, with a particular focus on emerging public health innovations and their policy implications.

## Methodology

This review aims to synthesize current knowledge on emerging public health strategies in malaria control, their policy implications, and the need for climate change adaptation. The methodology for this review involves a systematic and comprehensive approach to identify, evaluate, and synthesize relevant literature and data.

Literature Search.

## Search strategy

A systematic search was conducted across multiple electronic databases, including PubMed, Google Scholar, Scopus, and Web of Science. The search strategy was designed to capture a broad range of studies, reports, and reviews on malaria control, technological advancements, vector control methods, policy implications, and climate change adaptation. Key search terms included:

- ‘malaria control strategies’
- ‘innovative malaria interventions’
- ‘genomic surveillance malaria’
- ‘rapid diagnostic tests malaria’
- ‘genetic modification mosquitoes malaria’
- ‘biological control malaria vectors’
- ‘spatial repellents malaria’
- ‘climate change adaptation malaria’
- ‘policy implications malaria control’

## Inclusion criteria

- Peer-reviewed articles, reviews, and meta-analyses published in English from 2010 to December 2023.
- Studies focusing on technological advancements in malaria control, novel vector control methods, policy implications, and climate change adaptation.
- Reports and guidelines from reputable organizations such as the WHO, Centers for Disease Control and Prevention (CDC), and national malaria control programs.

## Exclusion criteria

- Articles not available in English.
- Studies published before 2010.
- Research focusing on malaria treatment or clinical management without relevance to control strategies or public health implications.

Data Extraction and Synthesis.

## Data extraction

Relevant data were extracted from selected articles using a standardized data extraction form. Extracted information included:

- Study design and methodology.
- Key findings related to malaria control innovations.
- Technological advancements in diagnostics and vector control.
- Policy recommendations and implications.
- Climate change impact and adaptation strategies.
- Recommendations for future research and policy.

## Data synthesis

The extracted data were systematically reviewed and synthesized to provide a comprehensive overview of current trends, innovations, and policy implications in malaria control. The synthesis focused on:

- Summarizing the latest technological advancements and their impact on malaria control.
- Evaluating the effectiveness and feasibility of novel vector control methods.
- Identifying key policy implications and recommendations for integrating new strategies into national and global malaria control programs.
- Assessing the need for and approaches to climate change adaptation in malaria control efforts.

## Quality assessment

Each selected study was assessed for quality and relevance using criteria adapted from established guidelines for systematic reviews. Criteria included:

- Study design and robustness of methodology.
- Relevance to the review’s objectives.
- Clarity and consistency of findings.
- Potential biases and limitations.

## Expert consultation

To complement the literature review, consultations with experts in malaria control, public health policy, and climate change adaptation were conducted. These consultations provided additional insights and perspectives on emerging strategies, policy challenges, and practical considerations for implementation.

## Reporting and review

The findings from the literature review and expert consultations were compiled into a comprehensive report. The report was structured to provide clear and actionable insights, with sections dedicated to technological advancements, novel vector control methods, policy implications, and climate change adaptation. The report was reviewed by external experts for accuracy and comprehensiveness before finalization.

## Technological advancements in malaria control

Technological advancements have significantly contributed to the arsenal of tools available for malaria control. These innovations encompass various areas, from surveillance and diagnosis to treatment and prevention. Genomic surveillance involves the sequencing and analysis of malaria parasite genomes to track their spread, detect drug-resistance mutations, and understand transmission dynamics^13^. By analyzing genetic data, researchers and public health authorities can identify emerging threats, tailor interventions, and monitor the effectiveness of control measures. Rapid diagnostic tests (RDTs) have revolutionized malaria diagnosis by enabling quick and accurate detection of malaria antigens in blood samples^10^. These tests are particularly valuable in resource-limited settings where access to laboratory facilities is limited. In addition to RDTs, molecular diagnostic techniques such as polymerase chain reaction (PCR) and LAMP offer higher sensitivity and specificity, allowing for the detection of low parasite densities and differentiation between species^11^. Advances in drug discovery technologies have led to the identification of novel antimalarial compounds and the development of more effective treatments^14^. High-throughput screening methods, structure-based drug design, and computational modeling techniques have accelerated the drug discovery process, resulting in the discovery of new drug candidates with novel mechanisms of action and improved safety profiles. The development of malaria vaccines represents a major milestone in malaria control efforts. The most advanced vaccine candidate to date is RTS,S/AS01 (Mosquirix), which has shown partial efficacy against malaria in clinical trials^15^. Other vaccine candidates targeting different stages of the parasite’s lifecycle are also under development, offering the potential for more effective protection against the disease.

Technological innovations have transformed vector control strategies with the development of new insecticides, formulations, and delivery systems. Long-lasting insecticidal nets (LLINs), insecticide-treated clothing, and spatial repellents offer alternative methods for reducing human-vector contact and preventing malaria transmission^16^. Moreover, genetic engineering technologies, such as gene drive systems, hold promise for modifying mosquito populations to reduce their ability to transmit malaria. Information and Communication Technologies (ICT) tools, including mobile phones, geographic information systems (GIS), and remote sensing technologies, play a crucial role in malaria surveillance, monitoring, and response^17^. Mobile health (mHealth) applications facilitate real-time data collection, reporting, and communication among healthcare workers, enabling timely interventions and decision-making. Data analytics and mathematical modeling techniques are increasingly being used to analyze malaria data, predict transmission patterns, and evaluate the impact of control measures^18^. Predictive models based on environmental factors, climate data, and vector biology help identify high-risk areas and optimize resource allocation for targeted interventions.

Point-of-care diagnostic tools and treatment devices enable healthcare providers to deliver timely and effective care to patients in remote and underserved areas. Portable devices for rapid diagnosis and drug administration improve access to healthcare services and reduce the burden on healthcare infrastructure^10^. Remote sensing technologies, such as satellite imagery and drones, provide valuable data for mapping vector habitats, monitoring environmental changes, and predicting malaria outbreaks. These tools enhance the effectiveness of vector control interventions and support decision-making in malaria control programs^17^. Digital health platforms and electronic health records facilitate data management, surveillance, and monitoring of malaria cases and interventions. Integrated information systems enable seamless coordination among stakeholders and support evidence-based decision-making at all levels of the healthcare system^18^.

## Implementation of technological advancements in malaria control

The implementation of technological advancements plays a pivotal role in advancing malaria control efforts worldwide. Technological advancements in insecticide development have led to the creation of next-generation compounds and formulations designed to combat insecticide resistance among malaria vectors. Development of insecticides with novel modes of action that target specific biochemical pathways in mosquitoes, reducing the likelihood of resistance. Techniques that prolong the effectiveness of insecticides, ensuring sustained mosquito mortality and reducing the frequency of reapplication. Utilization of spatial repellents that emit odors or chemicals to deter mosquitoes from human hosts or treated areas, reducing vector-human contact. Application of larvicides in breeding sites to prevent mosquito larvae from maturing into adults, complementing adult vector control methods like insecticide-treated nets (ITNs) and indoor residual spraying (IRS). Continued advancements in RDT technology for detecting malaria antigens with high sensitivity and specificity, enabling prompt diagnosis in remote and resource-limited settings. Integration of molecular techniques such as polymerase chain reaction (PCR) and loop-mediated isothermal amplification (LAMP) for detecting low parasite densities and differentiating between Plasmodium species. Utilization of Geographical Information Systems (GIS) for mapping malaria transmission hotspots, vector breeding sites, and environmental factors influencing disease spread. Deployment of mHealth tools for real-time data collection, reporting of malaria cases, and surveillance updates from remote locations to central databases, facilitating timely response and resource allocation. Research into new classes of antimalarial drugs with improved efficacy, safety profiles, and resistance management strategies, targeting both blood and liver stages of the malaria parasite. Development of innovative drug delivery systems including long-acting formulations, nanoparticles, and implants to ensure sustained therapeutic concentrations and improve patient adherence. Utilization of telemedicine for remote consultations, training of healthcare workers, and dissemination of educational materials on malaria prevention and treatment. Development of mobile applications featuring educational content, interactive quizzes, and SMS alerts to raise awareness and promote behavior change among at-risk populations. Advancing research and clinical trials for malaria vaccines, including RTS,S/AS01 (Mosquirix) and next-generation vaccine candidates, to provide long-term protection against Plasmodium infection. Exploration of genetically modified mosquitoes (e.g., gene drive technologies) to suppress vector populations or render them incapable of transmitting malaria parasites. Ensuring equitable access to technological innovations across diverse populations, including marginalized communities and remote regions. Establishing sustainable funding mechanisms and infrastructure to support the long-term deployment and maintenance of technology-driven interventions^15–18^.

## Novel vector control methods

Novel vector control methods are essential in the fight against malaria, especially as traditional approaches face challenges such as insecticide resistance and ecological concerns. Here are some innovative vector control methods that have emerged in recent years:

1. Genetic modification of mosquitoes: Genetic engineering technologies, including gene drive systems, offer the potential to modify mosquito populations to reduce their ability to transmit malaria. Gene drive technology enables the rapid spread of genetic modifications through mosquito populations, such as rendering mosquitoes incapable of transmitting the malaria parasite or reducing their reproductive capacity^19^. While still in the experimental stages, these approaches hold promise for sustainable and targeted vector control.
2. Biological control: Biological control methods utilize natural predators, parasites, or pathogens to suppress mosquito populations^20^. For example, the introduction of larvivorous fish species, such as Gambusia affinis, into mosquito breeding habitats can reduce mosquito larvae numbers. Similarly, the use of bacteria, fungi, or viruses that target mosquitoes, such as Bacillus thuringiensis israelensis (Bti) or Wolbachia, can disrupt mosquito reproduction or development.
3. Attractive toxic sugar baits (ATSB): ATSBs are bait stations containing a sugar solution laced with a small amount of insecticide^21^. These baits attract mosquitoes seeking sugar meals, particularly outdoor-resting malaria vectors. Once ingested, the insecticide kills the mosquitoes, reducing local vector populations. ATSBs offer a targeted and environmentally friendly approach to mosquito control, with potential applications in urban and peri-urban settings.
4. Spatial repellents: Spatial repellents release volatile compounds that repel mosquitoes from enclosed spaces, such as houses or outdoor gathering areas^22^. These repellents create a protective barrier against mosquito bites, reducing human-vector contact and malaria transmission. Unlike insecticides, spatial repellents do not necessarily kill mosquitoes but instead deter them from entering treated areas, offering a non-toxic alternative for vector control.
5. Targeted indoor residual spraying (TIRS): TIRS involves the application of insecticides to specific indoor surfaces where mosquitoes rest, rather than spraying entire rooms or houses^23^. By targeting mosquito resting sites, such as walls, ceilings, and indoor surfaces near human sleeping areas, TIRS maximizes insecticide effectiveness while minimizing environmental impact and insecticide exposure to non-target organisms.
6. Sterile insect technique (SIT): SIT involves the mass production and release of sterilized male mosquitoes into target areas to mate with wild females^24^. As sterile males do not produce viable offspring; repeated releases can suppress or eliminate local mosquito populations over time. SIT has been successfully used to control other insect pests and shows potential for malaria vector control, particularly in combination with other interventions.
7. Repellent-treated clothing: Repellent-treated clothing impregnated with insecticides or repellents provides personal protection against mosquito bites, particularly during outdoor activities or in areas with high malaria transmission^25^. These clothing items offer a convenient and long-lasting alternative to topical repellents, reducing the need for frequent reapplication and improving compliance with malaria prevention measures.
8. Environmental management: Environmental modification techniques aim to manipulate mosquito breeding habitats to reduce larval populations^26^. Strategies such as habitat manipulation, drainage, and larval source management target mosquito breeding sites, such as stagnant water bodies, to disrupt the mosquito lifecycle and reduce malaria transmission. These approaches are particularly effective when implemented as part of integrated vector management (IVM) strategies.
9. Insecticide resistance management: Innovative approaches to managing insecticide resistance include rotating or combining different classes of insecticides, using synergists to enhance insecticide efficacy, and developing new insecticide formulations with novel modes of action^27^. These strategies aim to prolong the effectiveness of existing insecticides and prevent the spread of resistance among mosquito populations.
10. Community engagement and empowerment: Community-based vector control approaches involve engaging local communities in mosquito control efforts, raising awareness about malaria transmission and prevention, and empowering community members to take ownership of vector control activities^28^. By involving communities in decision-making and implementation, these approaches foster sustainable behavior change and enhance the effectiveness of vector control interventions.
11. Vector control methods with practical and logistical challenges of implementing the strategies.
  Vector control methods are crucial in the fight against malaria, aiming to reduce mosquito populations and interrupt transmission cycles. Insecticide-treated nets (ITNs) are bed nets treated with insecticides, typically pyrethroids, which repel and kill mosquitoes that come into contact with them. They provide a physical barrier and reduce the likelihood of mosquito bites during sleeping hours when Anopheles mosquitoes are most active. Ensuring universal access to ITNs in malaria-endemic regions remains a challenge due to logistical issues in distribution and affordability for vulnerable populations. Over time, mosquitoes may develop resistance to insecticides used in ITNs, necessitating regular monitoring and potentially rotating to alternative insecticides. Compliance with consistent and correct use of ITNs can vary among communities, influenced by cultural practices, comfort preferences, and perceptions of efficacy^29–33^. Indoor residual spraying (IRS) involves spraying insecticides on the interior walls and ceilings of houses and structures where mosquitoes rest after feeding. This method targets mosquitoes that enter homes and reduces their lifespan upon contact with treated surfaces. Indoor residual spraying requires trained personnel for proper application, sufficient supplies of insecticides, and logistical coordination to cover targeted areas within specified timeframes. Acceptance of IRS can vary among communities due to concerns about odor, the safety of insecticides, and disruption to daily activities during spraying campaigns. Maintaining sustained coverage and effectiveness of IRS programs over time requires ongoing funding, infrastructure support, and community engagement^34–36^.
  Larval Source Management (LSM) involves targeting and treating mosquito breeding sites, such as stagnant water bodies, with larvicides or biological control agents to prevent larvae from maturing into adult mosquitoes. Identifying and mapping all potential breeding sites can be challenging, particularly in complex urban environments or remote rural areas. The application of larvicides must consider environmental impact and potential harm to non-target organisms in aquatic ecosystems. Maintaining regular monitoring and larvicide application requires ongoing resources and community involvement to ensure long-term effectiveness. Spatial repellents emit volatile compounds that deter mosquitoes from entering treated areas or approaching human hosts, reducing vector-human contact. Ensuring consistent and long-lasting efficacy of spatial repellents can be challenging, particularly in diverse environmental conditions and varying mosquito species. Community acceptance and willingness to adopt spatial repellents may vary based on perceived effectiveness, cultural preferences, and ease of application. Coordinating spatial repellents with other vector control methods requires strategic planning and assessment of synergistic effects. Limited funding and resources can constrain the scale-up and sustainability of vector control interventions, particularly in low-resource settings. Weak healthcare infrastructure and logistical challenges in transportation and distribution can hinder the timely delivery of insecticides, nets, and other supplies. Effective implementation requires robust monitoring and evaluation systems to assess coverage, compliance, and impact on malaria transmission. Engaging communities in the design and implementation of vector control strategies is essential for ensuring acceptance, sustainability, and adherence to recommended practices^37,38^.
12. Potential ecological impacts of gene drive technology

Gene drive technology holds promise for addressing vector-borne diseases like malaria by genetically modifying mosquito populations to reduce their ability to transmit diseases. However, it also raises significant ecological concerns due to its potential long-term impacts on ecosystems. Altering the population dynamics of target mosquito species could disrupt ecological interactions within ecosystems. For example reducing the population of a mosquito species might affect predators or parasites that depend on them for food. Decreasing the population of target mosquitoes could create ecological niches that allow other species to proliferate, potentially leading to changes in biodiversity and ecosystem stability. There is a risk that gene drive constructs could spread horizontally to non-target species or to other populations of the target species, especially if containment measures fail or if mosquitoes migrate to new areas. Once released into the environment, gene drives may spread rapidly and be challenging to control, potentially affecting ecosystems beyond the intended geographic or ecological boundaries. Target mosquito populations could develop resistance to the gene drive mechanism, leading to the emergence of new mosquito strains that are resistant to control efforts. This could complicate disease control strategies and require continual adaptation of gene drive technologies. Altered mosquito populations may adapt in unexpected ways, potentially influencing their behavior, life history traits, or habitat preferences. These adaptations could have cascading effects on ecosystem functions and services. Changes in insect populations, even if they are mosquitoes, could affect pollination patterns or seed dispersal mechanisms, potentially impacting plant diversity and agricultural productivity. Disruptions to mosquito populations could affect the food sources of other organisms in the ecosystem, such as insectivorous birds, bats, and fish, leading to cascading effects throughout food webs. Concerns over the ecological impacts of gene drive technology may influence public acceptance and willingness to support its implementation, potentially hindering efforts to combat vector-borne diseases. The distribution of benefits and risks associated with gene drive technologies may raise ethical concerns, particularly regarding vulnerable populations and communities in malaria-endemic regions. Conducting comprehensive ecological risk assessments before field trials and deployment to anticipate potential impacts and inform decision-making. Developing robust containment strategies to prevent the unintended spread of gene drive constructs beyond target populations, including gene editing reversibility and gene drive suppression technologies. Implementing rigorous monitoring and surveillance programs to track the spread and ecological impacts of gene-drive-modified organisms in the environment. Engaging stakeholders, including local communities, conservation groups, and regulatory agencies, in transparent discussions about the ecological risks and benefits of gene drive technologies^35–38^.

## Policy implications

The emergence of novel approaches and advancements in malaria control has significant policy implications at local, national, and global levels. Here are some key policy implications of these developments:

1. Integration into National Malaria Control Programs: Governments and health authorities need to incorporate emerging malaria control strategies into their national malaria control programs^29^. This integration should involve updating policies, guidelines, and protocols to reflect the latest evidence-based interventions and best practices. National malaria control strategies should prioritize the adoption of innovative vector control methods, diagnostic technologies, and treatment regimens to improve malaria prevention, diagnosis, and treatment outcomes.
2. Resource allocation and funding prioritization: Policymakers must allocate adequate resources and funding to support the implementation of emerging malaria control strategies^30^. This includes investment in research and development, capacity building, procurement of essential tools and commodities, and strengthening health systems to deliver quality malaria services. Governments should prioritize funding for innovative vector control methods, such as genetic modification of mosquitoes and spatial repellents, alongside traditional interventions like insecticide-treated nets and indoor residual spraying.
3. Regulatory frameworks and approval processes: The development and deployment of novel malaria control interventions may require regulatory approval and oversight to ensure safety, efficacy, and ethical considerations^31^. Policymakers need to establish regulatory frameworks and approval processes to assess the risks and benefits of new interventions, including genetic modification technologies and novel insecticides. These frameworks should involve consultation with relevant stakeholders, including scientists, public health experts, and community representatives, to facilitate informed decision-making.
4. Capacity building and training: Policymakers should invest in capacity building and training programs to equip healthcare workers, researchers, and vector control personnel with the skills and knowledge needed to implement emerging malaria control strategies effectively. This includes training in the use of new diagnostic technologies, genetic engineering techniques, and community engagement approaches. Capacity-building efforts should be tailored to the specific needs and contexts of different countries and regions, with a focus on building local expertise and sustainability.
5. Monitoring and evaluation: Robust monitoring and evaluation systems are essential for assessing the impact and effectiveness of emerging malaria control strategies^32^. Policymakers should establish surveillance systems to monitor malaria transmission, insecticide resistance, and the prevalence of malaria parasites. Monitoring and evaluation efforts should also assess the coverage, quality, and equity of malaria interventions, identify gaps and challenges, and inform programmatic adjustments and resource allocation decisions.
6. Global collaboration and partnerships: Addressing the complex challenges of malaria control requires global collaboration and partnerships among governments, international organizations, research institutions, civil society organizations, and the private sector^33^. Policymakers should foster collaboration and knowledge sharing to accelerate the development, deployment, and scale-up of innovative malaria control interventions. This includes sharing data, research findings, and best practices, as well as coordinating funding and technical support for malaria control efforts in endemic countries.
7. Advocacy and political commitment: Policymakers at all levels should advocate for political commitment and sustained investment in malaria control efforts^34^. This includes raising awareness among policymakers, donors, and the public about the importance of malaria elimination as a public health priority and the potential of emerging strategies to achieve this goal. Strong political commitment is essential for mobilizing resources, overcoming challenges, and driving progress towards malaria eradication.

## Climate change adaptation

Climate change adaptation is becoming increasingly important in the context of malaria control, as shifts in temperature, precipitation patterns, and ecological conditions impact the distribution and transmission of the disease. Policymakers must integrate climate data into malaria surveillance systems to anticipate and respond to changes in transmission patterns^35^. This involves using climate models and weather forecasts to predict malaria outbreaks, identify high-risk areas, and allocate resources for targeted interventions. Early warning systems can alert health authorities to impending outbreaks, enabling proactive measures to prevent transmission and reduce morbidity and mortality. Climate change influences the distribution, behavior, and abundance of mosquito vectors, altering malaria transmission dynamics^35^. Policymakers should adjust vector control strategies to address changing vector ecology and distribution patterns. This may include expanding the use of insecticide-treated nets (ITNs) and indoor residual spraying (IRS) to new geographic areas, implementing larval source management in response to shifting breeding habitats, and exploring novel vector control methods suited to changing environmental conditions.

Strengthening health systems is essential for building resilience to climate change impacts on malaria^37^. Policymakers should invest in health infrastructure, human resources, and supply chain management to ensure the continuity and effectiveness of malaria control services amidst environmental variability and extreme weather events. Capacity building and training programs can empower healthcare workers to respond effectively to climate-related health threats and emergencies. Engaging communities in climate change adaptation efforts is critical for enhancing resilience and sustainability^38^. Policymakers should promote community participation in malaria control programs, raise awareness about climate-related health risks, and empower local populations to take adaptive measures. Community-based approaches, such as community health worker programs and participatory decision-making processes, foster ownership and collaboration in adapting malaria control strategies to changing environmental conditions. Addressing the complex interplay between climate change and malaria requires collaboration across sectors, including health, environment, agriculture, water resources, and disaster management. Policymakers should promote intersectoral collaboration and coordination to mainstream climate change adaptation into broader development agendas. Integrating malaria control with climate change adaptation and mitigation strategies can enhance synergies, maximize co-benefits, and build holistic resilience to climate-related health risks^37^.

Policymakers should prioritize research and innovation in climate-resilient malaria control strategies^39^. Investing in interdisciplinary research on climate-health linkages, vector ecology, and adaptation measures can generate evidence-based solutions to address emerging challenges. Policymakers should support innovative approaches, such as climate-smart agriculture, ecosystem-based vector control, and remote sensing technologies, to enhance the effectiveness and sustainability of malaria control in a changing climate. Climate change is a global challenge that requires coordinated action and solidarity among nations^40^. Policymakers should advocate for international cooperation, funding, and technical support to assist vulnerable countries in adapting malaria control strategies to climate change. Global initiatives, such as the Green Climate Fund and the Adaptation Fund, provide opportunities for mobilizing resources and building capacity for climate resilience in malaria-endemic regions.

## Climate change adaptation plans already implemented in some countries

### Netherlands

A comprehensive system of dams, dikes, locks, and storm surge barriers to protect against sea-level rise and flooding. Redesigning riverbanks and floodplains to accommodate increased river discharge during heavy rainfall events. Integrating green roofs, permeable pavements, and water storage systems to manage urban flooding^35^.

### Bangladesh

Building protective embankments along vulnerable coastal areas and constructing flood shelters to provide refuge during cyclones and floods. Implementing community-based early warning systems to alert residents of impending disasters. Promoting climate-resilient crop varieties and techniques like raised-bed gardening to cope with changing rainfall patterns^36^.

### Australia

Investing in water conservation, recycling, and desalination plants to address water scarcity exacerbated by droughts. Enhancing bushfire preparedness and response strategies, including early warning systems and controlled burns. Retrofitting buildings and infrastructure to withstand extreme heatwaves and storm events^37^.

### Maldives

Protecting coral reefs and mangroves to act as natural barriers against sea-level rise and storm surges. Constructing elevated roads, buildings, and water supply systems to mitigate flooding in low-lying atolls. Investing in renewable energy sources such as solar and wind to reduce dependence on imported fossil fuels vulnerable to climate impacts.

### United States

Enhancing coastal defenses and levee systems to protect against storm surges and sea-level rise. Planting trees and using cool roofing materials to reduce heat islands in cities and mitigate heat-related health risks. Restoring wetlands, floodplains, and natural habitats to enhance biodiversity and buffer communities from extreme weather events.

## Specific examples of existing or proposed regulations for new technologies and political and bureaucratic challenges that may arise in integrating new control strategies

The U.S. Environmental Protection Agency (EPA) and the Food and Drug Administration (FDA) have regulatory oversight over genetically modified organisms (GMOs), including gene drive organisms. The EPA assesses environmental risks, while the FDA evaluates human and animal health impacts. The European Union’s regulatory framework for GMOs, under Directive 2001/18/EC, requires risk assessments for environmental impacts before any release of genetically modified organisms, including those utilizing gene drive technology. An international agreement under the Convention on Biological Diversity, aiming to ensure the safe handling, transport, and use of living modified organisms (LMOs), including gene drive organisms, to minimize potential risks to biological diversity. Many countries and international bodies have developed ethical guidelines and principles for the responsible use of new technologies, including gene editing and gene drive, involving considerations of equity, consent, and transparency in research and deployment. Different countries have varying regulatory frameworks for GMOs and gene drive technologies, leading to challenges in harmonization and international collaboration. Conducting robust risk assessments requires scientific expertise and resources, which may be lacking in some regulatory agencies, especially in low- and middle-income countries. Gene drive technologies often provoke public debate and concern over ecological, ethical, and social implications, influencing political decisions and regulatory approvals. Effective communication of scientific evidence, risks, and benefits to policymakers, stakeholders, and the public is crucial but challenging, requiring clear messaging and transparency^39,40^. Coordinating international efforts and agreements on the regulation and deployment of gene drive technologies requires diplomacy and consensus-building among diverse stakeholders with varying interests and priorities. Ensuring equitable access to new technologies and addressing concerns of affected communities, particularly in malaria-endemic regions, requires navigating geopolitical tensions and balancing global health priorities with local needs. Building regulatory and scientific capacity in countries with limited resources to effectively assess and monitor gene drive technologies is essential but resource-intensive. Establishing infrastructure for monitoring, surveillance, and enforcement of regulations poses logistical and financial challenges, particularly in remote and underserved regions. Introducing genetically modified mosquitoes with gene drive technology for malaria control in a country with diverse ecosystems and regulatory frameworks. Negotiating political support and regulatory approval across multiple government agencies and levels of governance, balancing public health benefits with environmental and ethical concerns. Navigating bureaucratic processes for environmental risk assessments, public consultations, and stakeholder engagement to build consensus and ensure compliance with regulatory requirements. Addressing community perceptions, ethical considerations, and socio-economic impacts through transparent communication, education campaigns, and inclusive decision-making processes^41,42^.

## Future direction

Future innovations in vector control will focus on overcoming insecticide resistance and enhancing efficacy against malaria-transmitting mosquitoes. Developing novel compounds and formulations that target specific biochemical pathways in mosquitoes, minimizing environmental impact and resistance development. Harnessing natural predators or genetically modifying mosquitoes to reduce vector competence and population size. Utilizing novel chemicals or odors to deter mosquitoes from human hosts or trap them efficiently. Continued improvement of RDTs and molecular diagnostic tools like LAMP for accurate detection of Plasmodium species. Implementing automated systems capable of processing large numbers of samples quickly, essential for surveillance and outbreak response. Integrating geospatial mapping, remote sensing, and predictive modeling to monitor vector distribution, parasite prevalence, and environmental factors influencing transmission. Research into novel classes of antimalarials with different modes of action to combat resistant strains. Long-acting formulations, implants, or nanoparticles that ensure sustained drug release and improve treatment adherence. Tailoring treatment regimens based on genetic factors of both the parasite and the host to optimize therapeutic outcomes^41,42^.

Utilizing smartphones and tablets for real-time reporting, data visualization, and decision-making at the point of care. Providing remote consultations, training, and support for healthcare workers in malaria-endemic regions. Harnessing large datasets to predict disease trends, optimize resource allocation, and evaluate intervention effectiveness. Engaging local leaders, community health workers, and educators to promote preventive measures such as bed nets, indoor spraying, and prompt treatment seeking. Tailoring messages to address cultural beliefs, misconceptions, and barriers to malaria prevention and control. Implementing strategies that incentivize community participation and adherence to malaria control measures. Advancing efforts towards a highly effective malaria vaccine to complement existing control measures. Continued exploration of natural compounds, bioengineering, and repurposing of existing drugs for antimalarial activity. Deepening understanding of mosquito behavior, genetics, and interactions with Plasmodium parasites to inform vector control strategies^43^.

## Conclusion

The evolution of innovative approaches in malaria control, the policy implications thereof, and the adaptation to climate change are critical elements in the ongoing fight against this persistent global health challenge. Technological advancements, such as genomic surveillance and diagnostic technologies, offer unprecedented opportunities to enhance surveillance, diagnosis, and treatment of malaria. These innovations must be integrated into national malaria control programs, supported by adequate funding, and guided by robust regulatory frameworks to ensure safety and efficacy. Furthermore, novel vector control methods, including genetic modification of mosquitoes and biological control, hold promise for reducing malaria transmission and overcoming challenges such as insecticide resistance. These approaches should be complemented by community engagement, capacity building, and cross-sectoral collaboration to maximize impact and sustainability. Moreover, as climate change continues to reshape malaria transmission patterns, policymakers must prioritize climate change adaptation in malaria control strategies. This involves integrating climate data into surveillance and response systems, adjusting vector control strategies to changing ecological conditions, and strengthening health systems to build resilience to climate-related risks.

The global implementation of climate change adaptation strategies represents a critical response to the increasingly evident impacts of climate variability and change. Across various regions and sectors, countries have taken proactive measures to build resilience, reduce vulnerabilities, and safeguard communities, ecosystems, and economies from the adverse effects of a changing climate. Through initiatives like flood protection infrastructure, sustainable water management, ecosystem restoration, and renewable energy adoption, tangible benefits have been realized. These include enhanced disaster preparedness, reduced economic losses from climate-related disasters, improved public health outcomes, and strengthened ecological resilience. However, while progress is notable, challenges persist. These include the need for increased funding, capacity building, and technological innovation, as well as ensuring equitable distribution of adaptation benefits, particularly for marginalized and vulnerable populations.

## Ethical approval

Not applicable.

## Consent

Not applicable.

## Source of funding

Not applicable.

## Author contribution

E.I.O. performed the following roles: conceptualisation, methodology, supervision, draft writing, editing and approval before submission.

## Conflicts of interest disclosure

The authors declare no conflicts of interest.

## Research registration unique identifying number (UIN)

Not applicable.

## Guarantor

The guarantor is Emmanuel Ifeanyi Obeagu.

## Data availability statement

Not applicable.

## Provenance and peer review

Not applicable.
